# Maternal immune conditions are increased in males with autism spectrum disorders and are associated with behavioural and emotional but not cognitive co-morbidity

**DOI:** 10.1038/s41398-020-00976-2

**Published:** 2020-08-14

**Authors:** Shrujna Patel, Russell C. Dale, Destanie Rose, Brianna Heath, Christine W. Nordahl, Sally Rogers, Adam J. Guastella, Paul Ashwood

**Affiliations:** 1 Autism Clinic for Translational Research, Brain and Mind Centre, Children’s Hospital Westmead Clinical School, Faculty of Medicine and Health, University of Sydney, Camperdown, NSW Australia; 2 Kids Neuroscience Centre, The Children’s Hospital at Westmead, Faculty of Medicine and Health, University of Sydney, Westmead, NSW Australia; 3 Department of Medical Microbiology and Immunology and MIND Institute, UC Davis, Davis, CA USA; 4 Department of Psychiatry and MIND Institute, UC Davis, Davis, CA USA

**Keywords:** Pathogenesis, Autism spectrum disorders, Pathogenesis, Autism spectrum disorders

## Abstract

Epidemiological and animal research shows that maternal immune activation increases the risk of autism spectrum disorders (ASD) in offspring. Emerging evidence suggests that maternal immune conditions may play a role in the phenotypic expression of neurodevelopmental difficulties in children with ASD and this may be moderated by offspring sex. This study aimed to investigate whether maternal immune conditions were associated with increased severity of adverse neurodevelopmental outcomes in children with ASD. Maternal immune conditions were examined as predictors of ASD severity, behavioural and emotional well-being, and cognitive functioning in a cohort of 363 children with ASD (*n* = 363; 252 males, 111 females; median age 3.07 [interquartile range 2.64–3.36 years]). We also explored whether these outcomes varied between male and female children. Results showed that maternal asthma was the most common immune condition reported in mothers of children with ASD. A history of maternal immune conditions (*p* = 0.009) was more common in male children with ASD, compared to female children. Maternal immune conditions were associated with increased behavioural and emotional problems in male and female children. By contrast, maternal immune conditions were not associated with decreased cognitive function. The findings demonstrate that MIA may influence the expression of symptoms in children with ASD and outcomes may vary between males and females.

## Introduction

Autism spectrum disorder (ASD) is a pervasive neurodevelopmental disorder that typically appears in childhood. ASD affects 1 in 54 people in the United States^1^ and this rate has been increasing over time. ASD is characterized by impairments in social communication and interaction, as well as the presence of repetitive and restricted behaviours or interests^2^. The clinical presentation, progression, and outcomes of ASD can be largely heterogeneous, posing difficulties for identification and treatment^3–5^. The causes of ASD are unknown; genetic, epigenetic, and environmental factors have all been implicated^6,7^.

Increasing evidence suggests that immune system dysregulation^8–11^, particularly maternal immune activation (MIA)^12–14^, is one factor that may be involved in the pathophysiology of ASD and other neurodevelopmental disorders. An extensive body of preclinical research has provided substantial evidence for the MIA hypothesis in ASD^15–18^. Human epidemiological studies also link chronic immune/inflammatory conditions in mothers, such as autoimmune conditions^19–26^, and asthma/allergic conditions^27–29^, with increased risk of ASD and other neurodevelopmental disorders in offspring. Cohort studies have shown that autoimmune conditions are more prevalent in mothers of children with ASD^23^, particularly in cases of ASD with combined developmental delay^24^ or regression^25^. Similarly, maternal asthma has been linked with increased risk of ASD with combined intellectual disability in offspring^29^. Maternal asthma and allergies have also been associated with increased social impairment symptoms in children with ASD^30^.

Autoimmune conditions, asthma, and allergies involve activation of inflammatory pathways and elevated levels of cytokines and chemokines^31–35^. It has been posited that prenatal exposure to inflammatory conditions interferes with neurodevelopment and foetal programming, leading to adverse outcomes in offspring^36,37^. Many cytokines and chemokines can cross the blood–brain barrier and the placenta, both potential avenues through which foetal development can be disrupted^38–40^. However, cytokines do not necessarily need to cross these barriers to elicit their effects; they can bind receptors at the placental interface leading to downstream effects on the placenta and foetus. The role of cytokines in the pathophysiology of ASD is further supported by elevated levels of pro-inflammatory cytokines and chemokines found at birth or during development in at least a subset of individuals with ASD that has been correlated with increased severity of ASD symptoms^41–47^.

Many animal studies have found that MIA predominantly affects males, compared to females, in the domains of repetitive behaviour^48,49^, motor development^50^, and learning and memory^51,52^. These findings are reflected in the incidence rate of ASD with four times more human males^1^ being affected and higher rates of repetitive behaviour are found in males, compared to females^53,54^. A recent study in humans also showed that the child’s sex, along with gestational timing of maternal inflammation (measured by elevated pro-inflammatory cytokines), can contribute to differential behavioural outcomes in the child^55^. While males appear to be more vulnerable to a maternal inflammation-mediated expression of neurodevelopmental difficulties, the mechanisms underlying this association remain unclear. Moreover, ASD was not specifically investigated in this study. Offspring sex likely interacts with other factors, such as the type of maternal activation (e.g. infection vs. chronic diseases such as autoimmunity, or asthma), the timing (i.e. early vs. late gestation), the duration (short vs. episodic/continuous exposures), and severity of the MIA, to produce differential behavioural outcomes in males and females^55–57^.

The existing evidence suggests a possible link between maternal immune conditions and the observed ASD phenotype in offspring. Phenotypic outcomes in response to the presence of maternal immune conditions may vary by offspring sex. In this study, we explored whether maternal immune conditions (autoimmune, asthma, and other chronic immune/inflammatory conditions) were associated with increased severity of adverse outcomes in a large, well-characterized cohort of preschool-aged children with ASD. We compared the severity of ASD, behavioural and emotional well-being, and cognitive functioning between male and female children to explore the role of offspring sex in following exposures to maternal immune conditions. We hypothesized that children whose mothers had a history of immune/inflammatory conditions would have increased severity of ASD and behavioural and emotional problems, along with decreased cognitive function. Notably, this sample contains a relatively large proportion of females (*n* = 111), allowing for examination of sex differences.

## Methods

### Participants

Participants included mothers and children who were enrolled through the Autism Phenome Project (APP) or Girls with Autism Imaging of Neurodevelopment (GAIN) study, conducted at the University of California Davis MIND Institute. The GAIN study has an identical study design to the APP and enriches the predominantly male APP cohort with additional female participants. The study protocols, including recruitment and behavioural assessments for the APP and GAIN studies, have been previously described in detail^58–60^. All assessment measures described below were conducted as part of the APP and GAIN study protocols during enrolment in these studies. Children with a community diagnosis of ASD were included in this study (*n* = 363; 252 males, 111 females; median age 3.07 [interquartile range 2.64–3.36 years]). This ASD diagnosis was confirmed upon enrolment using the Autism Diagnostic Interview-Revised^61^ and the Autism Diagnostic Observation Schedule (ADOS)^62^. The presence of Fragile X syndrome or other neurological (e.g. seizures), psychiatric, or medical conditions were considered exclusion criteria for all children. The administration of all diagnostic instruments was carried out by experienced clinicians at the MIND Institute. All participants were English speakers, ambulatory, and had no suspected vision or hearing problems. All participants were screened via a parental interview for current and past physical illness. This study was approved by the institutional review board at the University of California, Davis. Informed consent was obtained before participation.

### ASD phenotype and offspring outcomes

ASD severity was measured using ADOS calibrated severity scores (CSSs). CSSs were calculated for the subdomains of social affect (CSS-SA) and restricted and repetitive behaviour, as well as an overall CSS. The ADOS CSSs are based on a scale on 1–10, where 1 represents minimal evidence of ASD-related symptoms and 10 denotes a high severity of symptoms^63,64^.

Developmental performance and cognitive functioning were assessed using the Mullen Scales of Early Learning (MSEL). The MSEL has components for visual reception, fine motor, receptive language, and expressive language. A developmental quotient (DQ) was calculated as the average of the age equivalent subscale scores divided by the chronological age at the time of testing and multiplied by 100. DQs provide a consistent metric for cognitive measures and accommodate floor effects. The nonverbal DQ (NVDQ) includes the visual reception and fine motor subscales, while the verbal DQ (VDQ) includes the receptive and expressive language subscales.

The Child Behaviour Checklist (CBCL)^65^, a widely used and well-accepted measure of child psychopathology^66,67^, was used to assess behavioural and emotional problems in children. The CBCL is a parent-rated questionnaire which contains a list of 100 behavioural/emotional problem items that parents rate as: not true, somewhat or sometimes true, or very or often true of their children. The CBCL produces raw scores that were transformed into three summary T scores (standardized by age and sex): (a) Total behaviour; (b) Externalizing (delinquency, aggression) behaviour; and (c) Internalizing (withdrawal, somatic complaints, anxious/depressed) behaviour; these were analysed as continuous variables referred to as ‘scores’. In addition, we analysed children who had CBCL T scores >60, which is an established threshold of clinically significant level of concern^65^, referred to as ‘morbidity’.

### Maternal immune history

Family medical history data, including the presence of maternal autoimmune conditions, chronic allergic/atopic conditions such as asthma, allergies, and eczema, and other inflammatory diseases was evaluated via a physician interview with the participant’s parent. Physicians were provided with a list of immune/autoimmune/inflammatory conditions to make this determination. Based on this information, mothers (and their children) were classified into two groups: maternal immune (one or more of the above immune-related conditions) and maternal non-immune.

### Statistical analysis

Demographic characteristics of mothers and children in the maternal immune and maternal non-immune groups were compared using independent-samples *t* tests, Mann–Whitney *U* tests, and Chi-square tests, as appropriate. Generalized linear models (normal distribution) were used to investigate the effect of maternal immune status on the continuous CBCL, MSEL, and ADOS scores, generating *β* coefficients and 95% confidence intervals (CIs). Generalized linear models (binomial distribution with logit link) were used to investigate CBCL morbidity (T score > 60) on the Total, Internalizing, and Externalizing scales, generating odds ratios (ORs) and 95% CIs. All models were adjusted for maternal age at childbirth and offspring sex. In addition, generalized linear models (adjusted only for maternal age at childbirth) were used separately in males and females to evaluate whether sex played a role in outcome differences between children in the maternal immune and maternal non-immune groups. Participants with missing data for an outcome measure were excluded from the analysis for that measure. There was a variable percentage of missing data in each measure for the cohort: CBCL Internalizing 14%, CBCL Externalizing 12%, CBCL Total 23%, MSEL VDQ 3%, MSEL NVDQ 3%, MSEL DQ 2%, and ADOS all scales 3%. Statistical analyses were performed using IBM SPSS Statistics 26 and graphs were created using GraphPad Prism 8.

## Results

### Characteristics of the maternal immune and maternal non-immune groups

Of the total 363 mothers included in the study, immune conditions of any type were found in 99 (27.27%) mothers (maternal immune group). The remaining 246 (72.73%) mothers who did not report any immune conditions served as a comparison group (maternal non-immune group). There were no significant differences between the maternal immune and maternal non-immune groups in the level of maternal education, annual household income, maternal age, and offspring race (Table 1). The median age of children in the maternal immune group was slightly lower than those in the maternal non-immune group (2.98 vs. 3.15 years, *p* = 0.0133; Table 1).

**Table 1:** Demographic characteristics of the mothers and children in the maternal immune and maternal non-immune groups (*n* = 363).

	Full cohort	Maternal immune	Maternal non-immune	*p* value^a^
Number of participants, *n* (%)	363	99	264	
Mother’s education, *n* (%)^b^				0.644
High school graduate or less	26 (7.16)	4 (4.04)	22 (8.3)	
Technical/vocational	37 (10.19)	12 (12.12)	25 (9.47)	
Some college credit	59 (16.25)	19 (19.19)	40 (15.15)	
Associate degree	47 (12.95)	12 (12.12)	35 (13.26)	
Bachelor’s degree	96 (26.45)	29 (29.29)	67 (25.38)	
Professional, Master’s, or Doctorate degree	47 (12.95)	15 (15.15)	32 (12.12)	
Annual family income, *n* (%)^b^				0.730
≤$29 000	38 (10.4)	8 (8.08)	30 (11.36)	
$30,000–$49,000	44 (12.12)	15 (15.15)	29 (10.98)	
$50,000–$74,999	54 (14.88)	17 (17.17)	37 (14.02)	
$75,000–$99,999	59 (15.25)	18 (18.18)	41 (15.53)	
$100,000–$149,999	48 (13.22)	11 (11.11)	37 (14.02)	
≥$150,000	48 (13.22)	14 (14.14)	34 (12.88)	
Mother’s age at childbirth, mean (SD)	31.57 (5.35)	31.87 (4.87)	31.46 (5.53)	0.528
Offspring race, *n* (%)^b^				0.517
Asian	29 (7.99)	7 (7.07)	22 (8.33)	
Mixed	41 (11.29)	11 (11.11)	30 (11.36)	
Other	31 (8.54)	6 (6.06)	25 (6.87)	
White/Caucasian	229 (63.09)	71 (71.72)	158 (59.85)	
Offspring age, median (IQR)	3.07 (2.64–3.36)	2.98 (2.53–3.23)	3.15 (2.70–3.38)	0.013^c^
Offspring sex, *n* (%)				0.009
Male	252 (69.42)	79 (79.80)	173 (65.53)	
Female	111 (30.58)	20 (20.20)	91 (34.47)	

*SD* standard deviation, *IQR* interquartile range.

^a^*p* Values are reported for comparisons between the maternal immune and maternal non-immune groups using independent-samples *t* tests, Mann–Whitney *U* test, or Chi-square tests as appropriate.

^b^Percentages expressed as a fraction of the number of participants in each group. Percentages may not total 100 due to missing data for some variables.

^c^Mann–Whitney *U* test used for offspring age as data were not normally distributed.

**Fig. 1:**
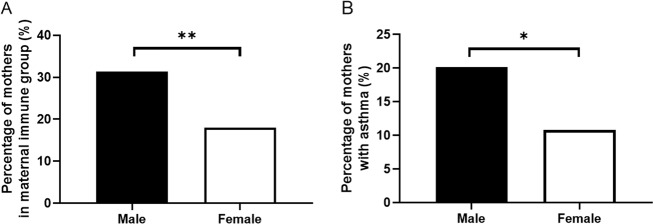
History of maternal immune conditions and asthma in male and female children with ASD. Percentage of mothers with any immune conditions (**a**) and asthma (**b**) were significantly higher in male children than female children.

Within the maternal immune group, asthma was the most common condition, found in 63 mothers (23.95% of total). Other frequent conditions included Hashimoto’s thyroiditis (*n* = 17), Raynaud’s disease (*n* = 10), alopecia (*n* = 5), psoriasis (*n* = 5), and rheumatoid arthritis (*n* = 4; Supplementary Table 1). Other immune conditions, each reported by 1–2 mothers in the immune group, are shown in Supplementary Table 1. A history of maternal immune conditions was more common in male children with ASD (31.3% of total) compared to female children (18.0%; *χ*^2^(1) = 6.904, *p* = 0.009; Fig. 1a). Specifically, a history of maternal asthma was twice as common in male children (20.2%) compared to female children (10.8%) with ASD (*χ*^2^(1) = 4.775, *p* = 0.029; Fig. 1b).

### Offspring outcomes

Maternal immune history was examined as a predictor of symptom outcomes in offspring by comparing the maternal immune and maternal non-immune groups. Owing to the high frequency of maternal asthma in the immune group, separate analyses were also conducted to examine maternal asthma alone as a predictor of offspring outcomes. In these analyses, mothers with asthma were compared with all mothers without asthma from both the maternal immune and maternal non-immune groups. The means and standard deviations of the CBCL, MSEL, and ADOS scores in the maternal immune and maternal non-immune groups are presented in Table 2.

**Table 2:** Descriptive statistics for offspring CBCL, MSEL, and ADOS scores in the maternal immune and maternal non-immune groups.

	Full cohort^a^	Maternal immune group	Maternal non-immune group
CBCL Internalizing, mean (SD)	62.05 (9.33)	62.95 (8.50)	61.72 (9.62)
CBCL Externalizing, mean (SD)	59.01 (10.61)	60.36 (10.47)	58.49 (10.65)
CBCL Total, mean (SD)	62.23 (10.54)	64.01 (9.81)	61.89 (10.76)
CBCL Internalizing >60, *n* (%)	184 (50.69)	56 (65.88)	128 (56.39)
CBCL Externalizing >60, *n* (%)	139 (38.29)	47 (53.41)	92 (40.00)
CBCL Total >60, *n* (%)	164 (45.18)	53 (69.74)	111 (54.41)
MSEL VDQ, mean (SD)	56.56 (25.25)	61.13 (24.81)	54.80 (25.25)
MSEL NVDQ, mean (SD)	69.74 (19.00)	75.11 (18.62)	67.68 (18.78)
MSEL DQ, mean (SD)	63.17 (20.64)	68.19 (19.61)	61.24 (20.74)
ADOS CSS-SA, mean (SD)	6.99 (1.67)	6.91 (1.77)	7.02 (1.64)
ADOS CSS-RRB, mean (SD)	8.42 (1.53)	8.30 (1.46)	8.46 (1.56)
ADOS CSS, mean (SD)	7.56 (1.72)	7.47 (1.77)	7.60 (1.70)

*SD* standard deviation, *CBCL* Child Behaviour Checklist, *MSEL* Mullens Scales of Early Learning, *VDQ* verbal developmental quotient, *NVDQ* nonverbal developmental quotient, *DQ* developmental quotient, *ADOS* Autism Diagnostic Observation Schedule, *CSS* calibrated severity score, *SA* social affect, *RRB* restricted repetitive behaviour.

^a^Percentage of missing data in each measure for the full cohort: CBCL Internalizing 14%, CBCL Externalizing 12%, CBCL Total 23%, MSEL VDQ 3%, MSEL NVDQ 3%, MSEL DQ 2%, ADOS all scales 3%.

Regarding behaviour, children in the maternal immune group had significantly higher CBCL scores on the Externalizing (*p* = 0.018) and Total (*p* = 0.021) scales (Table 3). When examining separately maternal asthma, there was also a significant association with increased Externalizing scores (*p* = 0.045) and risk of more impaired behaviour on the Externalizing scale (*p* = 0.007; Table 3). Maternal immune conditions were not associated with decreased cognition; significantly higher NVDQ (*p* = 0.029) and DQ (*p* = 0.001) scores were found on the MSEL assessment (Table 3). By contrast, there was no significant effect of maternal asthma alone and cognition on MSEL scores (Table 3). Regarding observational ADOS autism assessment, there were no significant effects of maternal immune conditions or maternal asthma on ADOS CSS scores (Table 3).

**Table 3:** General linear models showing maternal immune conditions and maternal asthma as predictors of offspring outcomes.

	Maternal immune group	Maternal asthma only
CBCL Internalizing, *β* [95% CI]	1.66 [−0.69, 4.01]; *p* = 0.166	2.28 [−0.48, 2.62]; *p* = 0.106
CBCL Externalizing, *β* [95% CI]	2.10 [−0.50, 4.71]; *p* = 0.113	3.09 [0.07, 6.12]; *p* = 0.045
CBCL Total, *β* [95% CI]	2.28 [−0.51, 5.07]; *p* = 0.109	2.45 [−0.84, 5.74]; *p* = 0.114
CBCL Internalizing >60, OR [95% CI]	1.70 [0.99, 2.92]; *p* = 0.54	1.71 [0.90, 3.24]; *p* = 0.104
CBCL Externalizing >60, OR [95% CI]	1.87 [1.11, 3.15]; *p* = 0.018	2.33 [1.26, 4.28]; *p* = 0.007
CBCL Total >60, OR [95% CI]	1.98 [1.11, 3.53]; *p* = 0.021	1.88 [0.94, 3.74]; *p* = 0.074
MSEL VDQ, *β* [95% CI]	2.03 [−0.45, 4.50]; *p* = 0.108	0.57 [−2.33, 3.48]; *p* = 0.699
MSEL NVDQ, *β* [95% CI]	6.75 [0.68, 12.82]; *p* = 0.029	1.48 [−5.67, 8.64]; *p* = 0.685
MSEL DQ, *β* [95% CI]	7.69 [3.18, 12.20]; *p* = 0.001	4.04 [−1.31, 9.39]; *p* = 0.139
ADOS CSS-SA, *β* [95% CI]	−0.13 [−0.53, 0.27]; *p* = 0.526	0.10 [−0.37, 0.57]; *p* = 0.668
ADOS CSS-RRB, *β* [95% CI]	−0.11 [−0.48, 0.27]; *p* = 0.579	−0.08 [−0.52, 0.36]; *p* = 0.721
ADOS CSS, *β* [95% CI]	−0.11 [−0.52, 0.32]; *p* = 0.625	0.11 [−0.38, 0.60]; *p* = 0.657

*OR* odds ratio, *CI* confidence interval, *CBCL* Child Behaviour Checklist, *MSEL* Mullens Scales of Early Learning, *VDQ* verbal developmental quotient, *NVDQ* nonverbal developmental quotient, *DQ* developmental quotient, *ADOS* Autism Diagnostic Observation Schedule, *CSS* calibrated severity score, *SA* social affect, *RRB* restricted repetitive behaviour.

The effects of maternal immune conditions on offspring outcomes, stratified by offspring sex, were next assessed (Table 4). Specifically, maternal immune conditions were associated with significantly increased Total CBCL scores (*p* = 0.026) and increased risk of impairment on the Externalizing scale (*p* = 0.045) in females (Table 4). Maternal asthma alone was associated with significantly increased risk of behavioural morbidity on the Externalizing scale in both males (*p* = 0.035) and females (*p* = 0.035; Table 4).

**Table 4:** General linear models showing maternal immune conditions and maternal asthma as predictors of offspring outcomes, separately in males and females.

	Maternal immune group		Maternal asthma only	
	Males only	Females only	Males only	Females only
CBCL Internalizing, *β* [95% CI]	1.15 [−1.46, 3.75]; *p* = 0.388	4.24 [−1.04, 9.52]; *p* = 0.116	2.34 [−0.66, 5.35]; *p* = 0.126	2.82 [−3.84, 9.48]; *p* = 0.406
CBCL Externalizing, *β* [95% CI]	1.98 [−0.91, 4.86]; *p* = 0.179	4.00 [−1.83, 9.83]; *p* = 0.179	2.53 [−0.76, 5.83]; *p* = 0.132	7.21 [0.01, 14.41]; *p* = 0.050
CBCL Total, *β* [95% CI]	1.42 [−1.63, 4.47]; *p* = 0.362	7.34 [0.88, 13.80]; *p* = 0.026	2.25 [−1.27, 5.77]; *p* = 0.210	5.20 [−3.37, 13.76]; *p* = 0.234
CBCL Internalizing >60, OR [95% CI]	1.45 [0.80, 2.62]; *p* = 0.219	4.83 [0.97, 24.00]; *p* = 0.054	1.53 [0.77, 3.04]; *p* = 0.232	4.41 [0.52, 37.61]; *p* = 0.175
CBCL Externalizing >60, OR [95% CI]	1.71 [0.95, 3.05]; *p* = 0.072	3.53 [1.03, 12.01]; *p* = 0.045	2.05 [1.05, 4.00]; *p* = 0.035	6.30 [1.14, 34.68]; *p* = 0.035
CBCL Total >60, OR [95% CI]	1.79 [0.96, 3.35]; *p* = 0.067	4.71 [0.87, 25.41]; *p* = 0.071	1.79 [0.86, 3.73]; *p* = 0.119	4.21 [0.43, 41.29]; *p* = 0.218
MSEL VDQ, *β* [95% CI]	0.21 [−2.55, 2.97]; *p* = 0.882	7.39 [2.08, 12.70]; *p* = 0.006	−1.30 [−4.52, 1.91]; *p* = 0.426	6.20 [−0.21, 12.60]; *p* = 0.058
MSEL NVDQ, *β* [95% CI]	4.00 [−2.90, 10.90]; *p* = 0.256	14.75 [1.93, 27.58]; *p* = 0.024	−1.28 [−9.33, 6.78]; *p* = 0.756	9.45 [−6.01, 24.91]; *p* = 0.231
MSEL DQ, *β* [95% CI]	5.66 [0.55, 10.76]; *p* = 0.03	13.49 [3.88, 23.10]; *p* = 0.006	2.54 [−3.46, 8.54]; *p* = 0.408	7.85 [−3.85, 19.56]; *p* = 0.188
ADOS CSS-SA, *β* [95% CI]	0.10 [−0.35, 0.56]; *p* = 0.664	−1.03 [−1.86, −0.20]; *p* = 0.015	0.32 [−0.21, 0.85]; *p* = 0.241	−0.76 [−1.76, 0.23]; *p* = 0.134
ADOS CSS-RRB, *β* [95% CI]	−0.11 [−0.54, 0.32]; *p* = 0.611	0.06 [−0.70, 0.83]; *p* = 0.875	0.08 [−0.42, 0.48]; *p* = 0.755	−0.49 [−1.39, 0.41]; *p* = 0.289
ADOS CSS, *β* [95% CI]	0.51 [−0.42, 0.54]; *p* = 0.815	−0.68 [−1.55, 0.20]; *p* = 0.129	0.33 [−0.23, 0.89]; *p* = 0.245	−0.67 [1.71, 0.37]; *p* = 0.207

*OR* odds ratio, *CI* confidence interval, *CBCL* Child Behaviour Checklist, *MSEL* Mullens Scales of Early Learning, *VDQ* verbal developmental quotient, *NVDQ* nonverbal developmental quotient, *DQ* developmental quotient, *ADOS* Autism Diagnostic Observation Schedule, *CSS* calibrated severity score, *SA* social affect, *RRB* restricted repetitive behaviour.

Females in the maternal immune group had significantly increased scores on all scales of the MSEL (VDQ *p* = 0.006, NVDQ *p* = 0.024, DQ *p* = 0.006; Table 4); however, no significant differences were found in males, suggesting that improvement was driven by females in this study. Maternal asthma alone did not have any significant effects on male or female MSEL scores (Table 4). In the maternal immune group, females showed a small, significant decrease in ADOS CSS-SA scores (*p* = 0.015; Table 4) suggesting better sociability, but male ADOS scores were not significantly affected. Maternal asthma alone did not significantly affect the ADOS scores in males or females (Table 4).

## Discussion

This study investigated whether maternal immune conditions (autoimmune, asthma, and other chronic immune/inflammatory conditions) influenced phenotypic outcomes in children with ASD. Further, we explored whether offspring sex interacts with the presence of maternal immune conditions to influence behavioural outcomes in children. In this cohort, a history of maternal immune conditions was more common in male children with ASD compared to female children. Maternal asthma, the most frequently reported immune condition, was also more common in male compared to female children. While previous studies have shown that maternal immune conditions are more prevalent in mothers of children with ASD^23–25^, our results suggest that this may be influenced by offspring sex, a finding not described previously. These results are consistent with animal research suggesting that males are more vulnerable to more neurodevelopmental abnormalities after MIA^48–52^.

Maternal immune conditions and maternal asthma were associated with increased CBCL scores and more impairment in children, particularly on the Externalizing and Total scales. The effect of maternal immune conditions was seen in female children only; however, the effect of maternal asthma was present in both male and female children. Overall, the results suggest that the presence of maternal immune conditions and maternal asthma may be linked to increased severity of behavioural and emotional problems in both male and female children with ASD.

These findings align with a recent study in humans which showed that elevated maternal cytokine levels in the first and second trimesters of pregnancy were associated with increased internalizing and externalizing symptoms in children. This study also found that gestational timing of inflammation and the child’s sex can influence behavioural outcomes^55^. The current study did not consider gestational timing; however, the immune conditions included here are chronic, rather than acute, in nature. Furthermore, the current study focussed specifically on behavioural outcomes in children with ASD, while the previous study explored outcomes in a general cohort. Maternal asthma and allergies have also been linked to increased social impairments in an Australian cohort studying children with ASD^30^, providing further support for the notion that maternal immune conditions, or in particular atopic/asthma immune activation, may influence behavioural and emotional outcomes in children in ASD. In a second large population-based cohort in the US, maternal asthma was associated with risk of ASD, with the children with ASD exhibiting more severe impairments^29^.

By contrast, maternal immune conditions were not associated with decreased cognitive functioning. Rather, maternal immune conditions were linked to higher NVDQ and DQ scores in the full cohort. However, this effect was not seen when examining the maternal asthma group separately. Furthermore, when analysed by sex, this improved cognition was only seen in females, who had higher scores on all three MSEL scales. These findings contrast with previous studies which showed that maternal autoimmune conditions were linked to ASD with combined intellectual disability and developmental delay^24,29^. However, these studies did not investigate male and female differences. The female specificity of high MSEL scores may demonstrate that, in cases of ASD where maternal immune conditions are present, female offspring are less likely to be susceptible to adverse cognitive outcomes in response to maternal inflammation than male offspring. Of note, animal studies support this hypothesis, showing that MIA detrimentally affects males, compared to females, in the domains of learning and memory^51,52^.

Of interest, the ADOS scores remained largely unaffected by maternal immune conditions, with only a small decrease found in females on the social affect domain. This finding is consistent with the notion that, in cases of ASD where maternal immune conditions are present, female children are less vulnerable to the detrimental effects of maternal inflammation. It is important to note that this cohort includes preschool-age children with an existing community diagnosis of ASD. It is possible that this sample is biased by children with more severe ASD as children with milder symptoms may not be diagnosed until a later age. Further studies, representing a broader spectrum of ASD, are required to determine whether maternal immune conditions influence the severity of ASD symptoms.

Overall, the results indicate that the presence of maternal immune conditions, particularly maternal asthma, may influence the phenotypic expression of ASD. In addition, offspring sex may contribute to the differential outcomes observed between male and female children in the maternal immune and maternal non-immune groups. Increasing evidence from preclinical models suggests that male offspring are more susceptible to adverse outcomes as a result of MIA compared to female offspring^48–52^. Although these models typically rely on short-term exposures to infections rather than potentially ongoing or episodic activation from chronic maternal immune conditions, the preclinical observations support findings seen in this study. While the findings of this study do not directly show greater impairments in male children, they do suggest that sex differences are an important factor in exposures from maternal immune conditions. Thus maternal immune conditions may be one environmental factor that contributes to the higher male prevalence seen in ASD.

The mechanisms underlying MIA and its conferred risk to offspring neurodevelopment are likely to be complex and multi-faceted. Epigenetic regulation of existing genetic vulnerabilities by MIA and other environmental factors likely places the foetus at greater risk for adverse outcomes^68–70^. Cytokines, chemokines, and antibodies, which are activated as a result of MIA, may interfere with foetal programming and development^36^. Microglia, the resident immune cells of the brain, have also emerged as key mediators of MIA^71–74^. Abnormalities in microglia phenotype, which can lead to disrupted synaptic pruning, have been associated with ASD and MIA^73,75,76^.

There are a number of potential limitations to this study. The data regarding maternal immune conditions was collected when children were enrolled in the study, not during pregnancy. It is possible that some mothers in the maternal immune group may not have had active immune conditions during gestation. The potential for recall bias in reporting notwithstanding, the presence of these conditions infers an immune dysregulation predisposition. Moreover, since the children were young at enrolment and given the chronic nature of the reported conditions, we suspect that underlying immune aberrations are likely to have been present in the mother during pregnancy^77–79^. Although there was a range of autoimmune conditions and other syndromes of inflammation reported in the maternal immune group, the majority of the mothers reported asthma. A further limitation of the study is that it is difficult to make inferences about many of the specific immune conditions other than asthma, especially those reported by only a few mothers. This study did not have data to directly investigate the influence of maternal infections during pregnancy, another important type of MIA, on child outcomes. Other variables that may contribute to inflammation or interact with existing conditions, such as obesity, diet, and stress, were also not collected^80,81^. There are other variables that could influence offspring outcomes which were not considered here, such as psychiatric conditions in mothers, recall bias in self-report of immune conditions, and use of medications during pregnancy.

The type and timing of MIA during pregnancy has been noted as an important factor which might exert differential effects on neurodevelopment^55,82,83^. Different types of MIA may utilize different immune molecules and pathways. For example, maternal asthma is characterized by T helper 2 (T_H_2) cell-mediated humoral responses^77,84^, whereas viral infections/bacterial infections are typically cell-mediated or T_H_1 responses^85^. Autoimmunity could be both T_H_1 and T_H_2, depending on the disease^34^. Innate immune responses, such as cytokine production, are also shared between asthma, autoimmunity, and infections^32,35^. In terms of the MIA animal models, different immune activators have been used to investigate different pathways, including the traditional infection-based model, as well as other pro-inflammatory states, such as asthma, obesity, and stress. However, based on this data, a clear finding was that immune activation caused by maternal asthma increases the risk for adverse offspring outcomes, providing clues to potential immune pathways that may be involved, such as the T_H_2 pathways.

Further research is warranted in humans to better understand the influence of maternal immune conditions on outcomes in children with ASD. Future studies should aim to comprehensively characterize immune activation in mothers during pregnancy, including the type, severity, and gestational timing of immune conditions, and then longitudinally examine offspring outcomes. Studies involving biomarkers and immunological profiling of both mothers and offspring are required to unravel the molecular mechanisms underpinning MIA. Such studies would enable clinical and biological characterization of immune-mediated subtypes in ASD. This may allow for the identification of therapeutic targets and exploration of immune-focussed medical treatments for subgroups of children with ASD. Furthermore, addressing mechanisms of maternal inflammation at the level of prenatal programming may lead to the development of prevention strategies for ASD.

This study shows that maternal immune conditions, particularly maternal asthma, are associated with increased behavioural and emotional problems in children with ASD. Furthermore, offspring sex may interact with maternal immune conditions to influence offspring outcomes in ASD, particularly in terms of cognition. This study adds to a growing body of literature which highlights that maternal immune conditions are an important factor in the phenotypic expression of ASD. Characterizing clinical associations between maternal immune conditions and symptom outcomes in children is an essential step in understanding the pathophysiology of ASD and has important implications for diagnosis and treatment.

## Supplementary information

Supplemental Table 1
