# Supplementary material for: Maternal immune conditions are increased in males with autism spectrum disorders and are associated with behavioural and emotional but not cognitive co-morbidity
Source: Transl Psychiatry. 2020 Aug 14;10:286. doi: 10.1038/s41398-020-00976-2 (PMC7429839; doi:10.1038/s41398-020-00976-2)
Supplement: Supplementary file 1 — Supplemental Table 1 [file 41398_2020_976_MOESM1_ESM.docx]

Supplementary Table 1: Frequencies of reported immune conditions by mothers in the maternal immune group (n = 99 of 363 participants).

| **Immune Condition** | **n (% of total cohort)** |
| --- | --- |
| Autoimmune conditions |  |
| Hashimoto’s thyroiditis | 17 (4.68) |
| Alopecia | 5 (1.38) |
| Psoriasis | 5 (1.38) |
| Rheumatoid Arthritis | 4 (1.10) |
| Lupus | 2 (0.55) |
| Type 1 diabetes | 2 (0.55) |
| Ulcerative colitis | 2 (0.55) |
| Antiphospholipid syndrome | 1 (0.28) |
| Crohn’s disease | 1 (0.28) |
| Grave’s disease | 1 (0.28) |
| Multiple sclerosis | 1 (0.28) |
| Rheumatic Carditis | 1 (0.28) |
| Sjogren’s syndrome | 1 (0.28) |
| Other autoimmune disease (not specified) | 1 (0.28) |
| Allergic/atopic conditions |  |
| Asthma | 63 (17.36) |
| Allergy | 2 (2.02) |
| Other immune/inflammatory conditions |  |
| Raynaud’s disease | 10 (10.10) |
| Idiopathic thrombocytopenic purpura | 2 (0.55) |
| Eosinophilic folliculitis | 1 (0.28) |
| Kawasaki disease | 1 (0.28) |
| Urticarial vasculitis | 1 (0.28) |
| White dot syndrome | 1 (0.28) |
